# Highly variable use of diagnostic methods for sexually transmitted infections-results of a nationwide survey, Germany 2005

**DOI:** 10.1186/1471-2334-10-98

**Published:** 2010-04-19

**Authors:** Andreas Gilsdorf, Alexandra Hofmann, Osamah Hamouda, Viviane Bremer

**Affiliations:** 1 Field Epidemiology Training Program (FETP), Berlin, Germany; 2 Department for Infectious Disease Epidemiology, Robert Koch-Institute, Berlin, Germany

## Abstract

**Background:**

Sexual transmitted infections (STIs) have increased in Germany and other countries in Europe since the mid-nineties. To obtain a better picture of diagnostic methods used in STI testing institutions in Germany, we performed a nationwide survey amongst STI specialists in order to evaluate the quality of STI reports and provide recommendations to harmonize and possibly improve STI diagnostics in Germany.

**Methods:**

We asked sentinel physicians and randomly chosen gynaecologists, urologists and dermato-venerologists, about the diagnostic methods used in 2005 to diagnose HIV, chlamydia (CT), gonorrhoea (GO) and syphilis (SY) in a national cross-sectional survey in order to recognize potential problems and provide recommendations.

**Results:**

A total of 739/2287 (32%) physicians participated. Of all participants, 80% offered tests for HIV, 84% for CT, 83% for GO and 83% for SY. Of all participants who performed HIV testing, 90% requested an antibody test, 3% a rapid test and 1% a nucleic acid amplification test (NAAT). For CT testing, NAAT was used in 33% and rapid tests in 34% of participants. GO resistance testing was performed by 31% of the participants. SY testing was performed in 98% by serology.

**Conclusions:**

Diagnostic methods for STI vary highly among the participants. Diagnostic guidelines should be reviewed and harmonised to ensure consistent use of the optimal STI diagnostic methods.

## Background

Most sexual transmitted infections (STIs) have increased in Germany and other countries in Europe in the mid-nineties [1-3], rising fears of subsequent increased HIV transmission. However, in the last couple of years not all STIs showed a similar epidemiology: While the number of new HIV and syphilis infections still rose in the last years [4-6], the number of newly diagnosed gonorrhoea infections declined in Europe [3]. Early detection and treatment are very important methods to control the transmission of STIs [7]. Since 2001, syphilis and HIV are the only notifiable STIs in Germany. Further data for STIs are collected through a sentinel surveillance system put in place in 2002 [8]. Thus, data for STIs such as HIV, syphilis, chlamydia, gonorrhoea and trichomoniasis is being collected from approximately 250 selected institutions nationwide. However, sentinel sites seemed to employ a variety of laboratory methods.

To obtain a better picture of diagnostic methods used in STI testing institutions in Germany, we performed a nationwide survey amongst the sentinel participants and other practicing STI specialists. This information will be used to evaluate the quality of STI reports and provide recommendations to harmonize and possibly improve STI diagnostics in Germany.

## Methods

We performed a national cross-sectional study. The study population included all sentinel sites (local health offices, hospital based STI clinics and private practitioners) participating in the STI sentinel surveillance plus randomly chosen private practitioners specialising in gynaecology, urology, or dermato-venerology. Lists of practitioners available from the state chambers of physicians were used for the random selection. We calculated the required sample size with StatCalc, EpiInfo6, with focus on the diagnosis on chlamydia. Assuming that 30% of the participants would employ NAAT for chlamydia testing this led to a calculated sample size requiring 620 participants. We expected a response of approximately one third of the contacted practitioners.

We developed a self-administered standardized questionnaire, with mainly closed questions, which was pretested before use. The questionnaire covered the different laboratory methods used for the detection of HIV, syphilis, chlamydia, gonorrhoea and trichimoniasis. We also asked information on the kind of samples taken (vaginal swab, urine, blood) and if asymptomatic patients were also tested for chlamydia, gonorrhoea or syphilis. In addition, the participants needed to provide the number and demographic characteristics of patients they see, as well as specify their type of catchment area (small or middle-sized cities, metropolitan etc).

In the first months of 2006 we asked sentinel sites and private practitioners to complete the questionnaire on laboratory methods used in the year 2005. This included both tests being performed in their own lab or sent away to external labs.

The laboratory methods and testing strategies for the following STIs were included in the questionnaire (see additional file 1), multiple answers were possible:

• HIV: Antibody test, rapid-test

• Chlamydia: Rapid test, DNA probe, nucleic acid amplification test (NAAT), antibody test; testing of asymptomatic patients

• Gonorrhoea: Microscopy, NAAT, DNA probe, culture; resistance patterns, testing of asymptomatic patients

• Trichimoniasis: Microscopy, culture

• Syphilis: Direct testing, dark field microscopy, serology; testing of asymptomatic patients

No further details on the specific tests were collected.

The completed questionnaires were entered into an MS Access database and analysed with EpiInfo 3.2.2. The results were stratified by medical speciality

## Results

Of 2287 contacted institutions and practitioners, 739 (32%) returned a questionnaire. Within the sentinel surveillance institutions, the response rate was 56%, while 30% of the randomly selected private practitioners responded.

A comparison between the sentinel and non-sentinel institutions participating did not show any major differences regarding the used diagnostic methods. Therefore, results are presented here not stratified by sentinel system participation.

In total, 219 dermato-venerologists, 355 gynaecologists, 85 urologists and 48 public health offices replied, 32 participants did not specify their specialisation. Fifty-two percent of the participants were situated in a metropolitan catchment area, 38% working in small or middle-sized cities. Participants replied from all federal states of Germany.

Chlamydia testing was offered by 621 participants (84%), followed by testing for syphilis (83%) and gonorrhoea (83%). Tests for HIV and trichimoniasis were offered by 592 (80%) participants and 575 (77%) respectively.

The proportions of participants testing for the different STIs, stratified by medical speciality, can be seen in Table 1.

**Table 1:** Proportions of participants testing for the different STIs in Germany in 2005, by medical speciality.

	Dermato-Venerology	Urology	Gynaecology	Public health offices
	n = 219	n = 85	n = 335	n = 48
	%	%	%	%
HIV	76	65	85	94
Chlamydia	72	84	95	67
Gonorrhoea	94	87	77	75
Trichonomas	63	79	95	44
Syphilis	90	68	82	92

### HIV

Of all participants who reported testing for HIV, 89% used antibody tests, 3% a rapid test and 1% NAAT. The remaining participants did not disclose the detection methods used. There were no differences between different medical specialisations.

### Chlamydia

The proportions of the different detection methods for chlamydia, stratified by medical speciality, are shown in Table 2.

**Table 2:** Proportions of the different detection methods for chlamydia used in Germany in 2005, by medical speciality.

	All	Dermato-Venerology	Urology	Gynaecology	Public health offices
	n = 621*	n = 159	n = 71	n = 337	n = 32
	%	%	%	%	%
Rapid test	**34**	44	17	48	6
DNA probe	**25**	22	34	26	16
NAAT (e.g. PCR)	**33**	34	38	29	56
Antigen test	**26**	26	25	28	13
Serology	**26**	38	31	19	16
Others	**1**	1	3	1	3

*Difference in overall sum due to lacking information of speciality of 22 participants testing on Chlamydia

Cervical swabs were the most frequently used samples for testing chlamydia (this varied depending on the method, median 77%). While gynaecologists relied almost exclusively on cervical samples (95%), urethral swabs were preferred by dermato-venerologists and urologists (89% resp. 81%).

### Gonorrhoea

For the detection of gonorrhoea, microscopy (64%) and culture (60%) were the most frequently used diagnostic tools.

Dermato-venerologists used microscopy 91% of the time, culture 56%. Gynaecologists performed cultures on 65% of samples and microscopy on 41%.

Samples for gonorrhoea examination were mainly urethral swabs (76%) followed by cervical swabs. Gynaecologists preferred cervical swabs (93%). Dermato-venerologists also performed regularly anal swabs (49%).

Testing for antimicrobial resistance was requested by 30% of the participants. Urologists most frequently requested resistance testing (47%), followed by gynaecologists (33%).

### Trichimoniasis

Trichimoniasis were examined in 94% of the participants by microscopy and in 22% with culture. There were no differences between the specialisations.

### Syphilis

Serology was used by 98% of the responding participants to look for syphilis. Serology was also used for confirmatory tests, to determine whether treatment is needed and for follow up. However, when asked for the different types of serological tests, participants' responses varied a lot. Eighteen point three percent of participants used dark field microscopy to identify syphilis, among dermato-venerologists this technique was frequent (42%). There were no further differences between the medical specialisations.

### Asymptomatic patients

Asymptomatic patients were tested for Chlamydia and syphilis by 65% and 65% of the participants, respectively. Antenatal screening was the main reason for these tests. Also, 33% of the participants reported testing of asymptomatic patients for gonorrhoea, giving "infection in the patient's partner" or "sexual risk behaviour" (unprotected sex or frequent changing of sex partners) as the main reasons for testing (Table 3).

**Table 3:** Reasons for testing asymptomatic patients for chlamydia, gonorrhoea and syphilis, Germany 2005.

	Chlamydia (n = 387)		Gonorrhoea (n = 188)		Syphilis (n = 376)	
	**n**	**%**	**n**	**%**	**n**	**%**
Partner infection	68	18	71	38	47	13
Antenatal care	205	53	18	10	170	45
Unspecific fluor	10	2	7	4	-	-
Skin diseases	-	-	-	-	38	10
Risk behaviour	31	8	44	23	63	17
Others	73	19	48	25	58	15

## Discussion

The results show that STI diagnostic methods varied a lot among the participants all over Germany. In addition, STI detection methods varied substantially between the different medical specialists. Antimicrobial resistance testing and testing of asymptomatic patients also differed considerably between participants. This could be seen also for sample materials; however a lot of this variation is self-evident, as the different specialists take mainly samples relevant for their work field (e.g. gynaecologists - vaginal swabs, urologists - urethral swabs).

The overall response rate was with 32% as expected. However the low percentage might have influenced the results, as it is possible that practitioners who responded to the survey are generally more aware of STIs and therefore more likely to offer comprehensive STI testing and adhere to testing guidelines. In reality, it is possible that fewer practitioners might offer STI testing and the test methods used may be even more diverse than shown by this survey. As participants did not perform all the testing themselves, but also used external laboratories, details on the reported used methods might be imprecise. However, we believe that the specialists mainly could give a good picture of the used methods.

### HIV

Nearly all participants used the same recommended test scheme for HIV [9], an Elisa screening test with sensitivity higher than 99%, followed by a Western blot as a confirmatory test with high specificity [10]:. But rapid tests become more relevant as it reflects on the strong wish of patients to have the results ready on the same day [10].

### Chlamydia

The best diagnostic method for chlamydia is NAAT [11] because of the high sensitivity (90-95%) and specificity (up to 100%), even though the appearance of a new variant of *Chlamydia trachomatis *challenged the NAAT test methods[12]. Our survey showed that only public health offices widely used NAAT.

Most participants followed the current recommendations [11] regarding sampling for chlamydia testing and took cervical or urethral swabs. Studies [13,14] showed that the first morning urine from men or self-collected vaginal swabs from women also provide good test results. In addition, these collection methods are less invasive, require less equipment and are well accepted by patients. However, the participants seldom requested these samples of the patients. Clinical practitioners need to be more widely informed on the advantages of using them.

The DNA probe test requires less complex transport, and is cheaper than NAAT, but is also less sensitive (60-80%) and specific (99.5%) [11,14]. In settings with few resources or those unable to access transport facilities, the DNA probe could be preferred.

Rapid tests for chlamydia, which show results within a very short time and require little technical effort, were used frequently. However, some studies show a considerably lower sensitivity and specificity compared to traditional detecting methods [11,15]. So the use of rapid tests cannot currently be recommended for routine diagnosis of chlamydia [15], but may be of use in environments with a population at high risk for the disease and where immediate treatment is preferable because of unlikely follow-up [10].

Serological testing for specific antibodies is not recommended for detection of acute uro-genital chlamydia infections, as many infections do not lead to a serological response [16]. However, it might be a useful tool to diagnose chlamydia-associated secondary complications in the absence of chlamydia symptoms, such as tubar sterility, reactive arthritis or Reiter-syndrome.

Since 2008, chlamydia screening has been introduced in Germany for a certain age group: all women under 25 years of age are offered a chlamydia test when visiting a gynaecologist [17]. Pooled urine samples are tested using NAAT. Though the use of pooled samples in the context of screening is perhaps controversial, this programme allows for the detection and treatment of asymptomatic infections in a higher proportion of young women than previously.

But women over the age of 25 years also need access to reliable methods for diagnosing chlamydia infections, as up to 80% of infections in these women are asymptomatic [18] and can lead to severe complications if untreated. Therefore, chlamydia testing using NAAT should be covered by all health insurances, regardless of the age of the patient.

### Gonorrhoea

Microscopy and culture are still the recommended method for the detection of gonorrhoea [19-21], even though NAAT is more sensitive [14,20], and could be used to detect different agents in the same reaction in urine specimens [22]. But *N. gonorrhoeae *species continues to present a considerable challenge for molecular diagnostics [21] and does not allow resistance testing. As antimicrobial resistance has increased recently [23,24], resistance testing on gonorrhoea isolates is recommended [21]. The number of participants performing antimicrobial resistance testing in our study was clearly inadequate, which raises concerns as infections with antibiotic-resistant strains will remain undetected, allowing spread of infections caused by antimicrobial-resistant gonorrhoea.

### Trichimoniasis

The most common and recommended method for detection of trichimoniasis is microscopy [25]. This is reflected in our results. Microscopy shows immediate but slightly unreliable results with sensitivity varying between 36 and 80% [14]. Culture before microscopy can increase the sensitivity [19]. Until NAAT for *Trichimonas vaginalis *is commercially availably, a stepwise approach using an additional rapid test for microscopy-negative women should be considered for adolescent women regardless of clinical factors [25].

### Syphilis

A vast majority of the participants used serological tests for the different levels of syphilis diagnosis. However, test methods were not used consistently. The availability of various diagnostic methods using different serological markers led to high variety in testing. NAAT methods became more relevant in recent years and can be used for diagnostic of primary syphilis in STI outpatient clinics and by general practitioners, but have no value for the diagnostic of secondary syphilis [26]. Clearer guidelines indicating the tests which should be used for different purposes are necessary to harmonize the diagnostics of syphilis in Germany.

### Asymptomatic patients

A high percentage of participants also offered syphilis and chlamydia testing for asymptomatic patients. Most of these tests were performed during mandatory antenatal screening. The guidelines regarding medical care during pregnancy [27], formulated by the German Federal Joint Committee require a pooled urine test for chlamydia and a serology test for syphilis during the first visits to a gynaecologist during pregnancy. Outside of antenatal care asymptomatic tests should be offered especially to persons reporting frequent changing of sexual partners or unprotected sexual contact.

### Monitoring diagnostic trends

Monitoring trends in the use of diagnostic methods enhances STI control efforts by improving the interpretation of STI trends, creating valuable partnerships with laboratories, facilitating the evaluation of screening programs and investigating outbreaks [28]. With the continuous development and improvement of diagnostic methods it is obvious that there have been changes since this survey was performed. But even though the presented results give a picture of the used methods in 2005, it is a relevant step for a better understanding of the changes that STI diagnostics are undergoing and its relevance for the interpretation of STI surveillance data.

## Conclusions

Our results showed dramatically inconsistent use of STI diagnostic methods across Germany. These methods should be harmonised to ensure comparable, high quality STI testing. Physicians require evidence-based information on the strengths and weaknesses of different diagnostic methods which will allow them to make an informed choice of the most appropriate tests, to correctly evaluate the results and therefore optimise treatment.

To achieve this, education on current standard diagnostic tools should be part of continuous professional education. Additionally, the physicians should be provided with harmonized, regularly updated guidelines from STI associations to ensure reliable, coordinated and high quality diagnosis and treatment of sexual transmitted diseases in Germany.

## Competing interests

The authors declare that they have no competing interests.

## Authors' contributions

AG und VB designed the study and the protocol. They were responsible for implementation of the survey. AH was strongly involved in data collection and analysis. AG produced the first draft of the paper, which was revised by VB. OH contributed to the protocol and survey, and revised the final paper. All authors have read and approved the final manuscript.

## Pre-publication history

The pre-publication history for this paper can be accessed here:

http://www.biomedcentral.com/1471-2334/10/98/prepub

## Supplementary Material

Additional file 1 **STI diagnostics questionnaire**. This additional file contains the questionnaire (in German) that was sent to the participants of the survey to collect the information on the different methods used for STI diagnostics in Germany in 2005. Click here for file
