# Supplementary material for: Highly variable use of diagnostic methods for sexually transmitted infections-results of a nationwide survey, Germany 2005
Source: BMC Infect Dis. 2010 Apr 19;10:98. doi: 10.1186/1471-2334-10-98 (PMC2873557; doi:10.1186/1471-2334-10-98)
Supplement: Additional file 1 — STI diagnostics questionnaire. This additional file contains the questionnaire (in German) that was sent to the participants of the survey to collect the information on the different methods used for STI diagnostics in Germany in 2005. [file 1471-2334-10-98-S1.PDF]

ROBERT KOCH INSTITUT

Befragung zur Diagnostik von sexuell übertragbaren Erkrankungen (STD)

Bitte zurücksenden an:

Dr. med. Andreas Gilsdorf
STD-Diagnostik
Abt. für Infektionsepidemiologie
Robert Koch-Institut
Seestraße 10
13353 Berlin

Stempel des/der Berichtenden (freiwillig):

Ausgefüllt durch Herrn/ Frau: ___________________________
(freiwillig, bitte Nichtzutreffendes streichen)

Bundesland: ________________________________
(Bitte auch bei anonymer Antwort ausfüllen)

Datum: ____

Befragung zur STD-Diagnostik
(Bitte beziehen Sie sich bei allen Antworten auf das Jahr 2005)

HIV

Führen Sie Untersuchungen auf HIV durch? Ja ☐1 Nein ☐0

Falls ja, nutzen Sie zur HIV-Diagnostik…

- einen HIV-Antikörper-Nachweis im Screening-Test (z.B. ELISA) gesichert durch Bestätigungstest (z.B. Western Blot, Immunfluoreszenztest)?
  Ja ☐1 Nein ☐0 Weiß nicht ☐9
  ↳ *falls ja,* bei wie viel Prozent der Untersuchungen? _ _ _ %
- einen HIV-Schnelltest? Ja ☐1 Nein ☐0 Weiß nicht ☐9
  ↳ *falls ja,* bei wie viel Prozent der Untersuchungen? _ _ _ %
- sonstiges: ____________________________, bei wie viel Prozent der Untersuchungen? _ _ _ %

Chlamydien-Infektion

Führen Sie Untersuchungen auf Chlamydien durch? Ja ☐1 Nein ☐0

Falls ja, nutzen Sie zur Diagnostik einer akuten Chlamydien-Infektion…

- einen Schnelltest? Ja ☐1 Nein ☐0 Weiß nicht ☐9
  ↳ *falls ja,* bei wie viel Prozent der Untersuchungen? _ _ _ %
- eine Gensonde? Ja ☐1 Nein ☐0 Weiß nicht ☐9
  (spezifisch markierte DNA/RNA-Sequenz) ↳ *falls ja,* bei wie viel Prozent der Untersuchungen? _ _ _ %
- einen Amplifikationstest? Ja ☐1 Nein ☐0 Weiß nicht ☐9
  (z.B. PCR, SDA,TMA) ↳ *falls ja,* bei wie viel Prozent der Untersuchungen? _ _ _ %
- einen Antigen-Nachweis? Ja ☐1 Nein ☐0 Weiß nicht ☐9
  (z.B. Enzym-Immuno-Assay, DFA) ↳ *falls ja,* bei wie viel Prozent der Untersuchungen? _ _ _ %
- einen Antikörper-Nachweis? Ja ☐1 Nein ☐0 Weiß nicht ☐9
  (Serologie) ↳ *falls ja,* bei wie viel Prozent der Untersuchungen? _ _ _ %
  ↳ *falls ja,* Methode: KBR ☐1 MIF ☐2 EIA ☐3 Westernblot ☐4
  sonstige ☐5, welche: ________________________
- sonstiges: ____________________________, bei wie viel Prozent der Untersuchungen? _ _ _ %

Bitte wenden→

Führen Sie auch bei asymptomatischen Patienten Untersuchungen auf Chlamydien durch?

- Ja ☐1 Nein ☐0 Weiß nicht ☐9

↳ *falls ja*, aus welchem Anlass? ____

____

Kreuzen Sie bitte an, bei welcher Methode Sie welche Probe auf Chlamydien untersuchen:

	Schnelltest	Gensonde	Amplifikationstest (z.B. PCR, SDA, TMA)	Antigentest (z.B. EIA, DFA)
Cervixabstrich				
Urin				
Vaginalabstrich				
Urethralabstrich				
Analabstrich				
Pharyngealabstrich				

Gonorrhö

Führen Sie Untersuchungen auf Gonorrhö durch? Ja ☐1 Nein ☐0

Falls ja, nutzen Sie zur Diagnostik einer akuten Gonorrhö …

- einen <u>mikroskopischen</u> Nachweis? Ja ☐1 Nein ☐0 Weiß nicht ☐9
  (nach Methylen-Blau/Gram-Färbung) ↳*falls ja,* bei wie viel Prozent der Untersuchungen? _ _ _ %
- einen <u>Amplifikationstest</u>? Ja ☐1 Nein ☐0 Weiß nicht ☐9
  (z.B. PCR, SDA, TMA) ↳ *falls ja,* bei wie viel Prozent der Untersuchungen? _ _ _ %
- eine <u>Gensonde</u>? Ja ☐1 Nein ☐0 Weiß nicht ☐9
  (spezifisch markierte DNA/RNA-Sequenz) ↳*falls ja,* bei wie viel Prozent der Untersuchungen? _ _ _ %
- eine <u>Kultur</u>? Ja ☐1 Nein ☐0 Weiß nicht ☐9
  ↳ *falls ja*, bei wie viel Prozent der Untersuchungen? _ _ _ %
- sonstiges: ______________________, bei wie viel Prozent der Untersuchungen? _ _ _ %

Führen Sie auch Antibiotika-Resistenzbestimmungen bei Gonorrhö durch?

- Ja ☐1 Nein ☐0 Weiß nicht ☐9

↳ *falls ja,* bei wie viel Prozent der Untersuchungen? _ _ _ %

Führen Sie auch bei asymptomatischen Patienten Untersuchungen auf Gonorrhö durch?

- Ja ☐1 Nein ☐0 Weiß nicht ☐9

↳ *falls ja*, aus welchem Anlass? ____

____

Kreuzen Sie bitte an, bei welcher Methode Sie welche Probe auf Gonorrhö untersuchen:

	Mikroskopischer Nachweis	Amplifikationstest (z.B. PCR, SDA, TMA)	Gensonde	Kultur
Cervixabstrich				
Urin				
Vaginalabstrich				
Urethralabstrich				
Analabstrich				
Pharyngealabstrich				

Trichomonas vaginalis

Führen Sie Untersuchungen auf Trichomonaden durch? Ja ☐1 Nein ☐0

Falls ja, nutzen Sie zur Diagnostik einer Trichomonaden-Infektion …

- eine mikroskopische Methode? Ja ☐1 Nein ☐0 Weiß nicht ☐9
  ↳ *falls ja,* bei wie viel Prozent der Untersuchungen? _ _ _ %
- eine Kultur? Ja ☐1 Nein ☐0 Weiß nicht ☐9
  ↳ *falls ja,* bei wie viel Prozent der Untersuchungen? _ _ _ %
- sonstiges: ____________________________, bei wie viel Prozent der Untersuchungen? _ _ _ %

Syphilis

Führen Sie Untersuchungen auf Syphilis durch? Ja ☐1 Nein ☐0

Falls ja, nutzen Sie zur Diagnostik einer Syphilis …

- einen Treponema-Direktnachweis? Ja ☐1 Nein ☐0 Weiß nicht ☐9
  (mittels monoklonaler Antikörper) ↳ *falls ja,* bei wie viel Prozent der Untersuchungen? _ _ _ %
- eine Dunkelfelduntersuchung? Ja ☐1 Nein ☐0 Weiß nicht ☐9
  ↳ *falls ja,* bei wie viel Prozent der Untersuchungen? _ _ _ %
- einen serologischer Nachweis? Ja ☐1 Nein ☐0 Weiß nicht ☐9
  ↳ *falls ja,* bei wie viel Prozent der Untersuchungen? _ _ _ %
  - Erfolgt der Suchtest mittels
    - TPHA/TPPA Ja ☐1 Nein ☐0 Weiß nicht ☐9
    - EIA Ja ☐1 Nein ☐0 Weiß nicht ☐9
    - VDRL/Cardiolipin Ja ☐1 Nein ☐0 Weiß nicht ☐9
  - Erfolgt der Bestätigungstest mittels
    - FTA-Abs Ja ☐1 Nein ☐0 Weiß nicht ☐9
    - IgG-Immunoblot Ja ☐1 Nein ☐0 Weiß nicht ☐9
    - EIA Ja ☐1 Nein ☐0 Weiß nicht ☐9
    - TPHA/TPPA Ja ☐1 Nein ☐0 Weiß nicht ☐9
  - Erfolgt die Beurteilung der Behandlungsbedürftigkeit mittels
    - 19-S-IgM-FTA-Abs-Test Ja ☐1 Nein ☐0 Weiß nicht ☐9
    - IgM-EIA Ja ☐1 Nein ☐0 Weiß nicht ☐9
    - IgM-Immunoblot Ja ☐1 Nein ☐0 Weiß nicht ☐9
    - VDRL/Cardiolipin Ja ☐1 Nein ☐0 Weiß nicht ☐9
  - Erfolgt die Verlaufskontrolle mittels
    - TPHA/TPPA Ja ☐1 Nein ☐0 Weiß nicht ☐9
    - 19-S-IgM-FTA-Abs-Test Ja ☐1 Nein ☐0 Weiß nicht ☐9
    - IgM-EIA Ja ☐1 Nein ☐0 Weiß nicht ☐9
    - VDRL/Cardiolipin Ja ☐1 Nein ☐0 Weiß nicht ☐9

Führen Sie auch bei asymptomatischen Patienten Untersuchungen auf Syphilis durch?

- Ja ☐1 Nein ☐0 Weiß nicht ☐9
  ↳ *falls ja*, aus welchem Anlass? ____

____

ROBERT KOCH INSTITUT

Angaben zur Praxis

Charakterisierung der Praxis

- Welche Facharztqualifikation haben Sie?

Dermatologie/Venerologie	☐ 1	Gynäkologie	☐ 4
Innere Medizin	☐ 2	Andere Fachrichtung	☐ 5
Urologie	☐ 3	Welche? ____________________	

- Wie stufen Sie Ihre Praxis von der Größe her ein?
  (Zahl der Scheine/Quartal; bei Praxisgemeinschaft nur Ihre Scheine)

<500	☐ 1	1000-1250	☐ 4
500-750	☐ 2	1250-1500	☐ 5
750-1000	☐ 3	>1500	☐ 6

- Wie würden Sie das Versorgungsgebiet Ihrer Praxis charakterisieren?

Ländlich/dörflich	☐ 1
Klein- bis mittelstädtisch	☐ 2
Großstädtisch	☐ 3

STD/HIV-Bereich

- Wie häufig nutzen Sie für die STD/HIV-Diagnostik Ihrer Patienten folgende Institutionen?

Eigene Laboreinrichtung	_ _ _ %	Landesuntersuchungsamt	_ _ _ %
Niedergelassener Laborarzt	_ _ _ %	Andere	_ _ _ %
Krankenhauslabor/Universität	_ _ _ %	Welche? ________________	

- Wie hoch schätzen Sie unter all Ihren Patienten den Anteil der

  STD-Patienten? _ _ _ %

  HIV-Patienten? _ _ _ %

- Wie viele Personen sind im <u>letzten Quartal</u> in Ihrer Praxis auf folgende STDs untersucht und diagnostiziert worden (nur Erstdiagnosen)?

STD	Anzahl Untersuchungen	Davon Positiv
HIV		
Chlamydien		
Gonorrhoe		
Syphilis		

Vielen Dank für Ihre Teilnahme!
